# Living bioethics, clinical ethics committees and children's consent to heart surgery

**DOI:** 10.1177/14777509211034145

**Published:** 2021-07-30

**Authors:** Priscilla Alderson, Deborah Bowman, Joe Brierley, Martin J. Elliott, Romana Kazmi, Rosa Mendizabal-Espinosa, Jonathan Montgomery, Katy Sutcliffe, Hugo Wellesley

**Affiliations:** 1 Social Research Institute, 4919 University College London, UK; 2 Medical Ethics, 156611 St George’s Hospital, UK; 3 Great Ormond Street Children's Hospital, London, UK; 4 Cardiothoracic Surgery, University College London, UK; 5 Chaplaincy, Great Ormond Street Hospital for Children NHS Foundation Trust, UK; 6 Faculty of Law, University College London, UK

**Keywords:** Clinical ethics, care for specific groups, minors, healthcare, healthcare quality, human experimentation, informed consent, incompetents, professional ethics in medicine

## Abstract

This discussion paper considers how seldom recognised theories influence clinical ethics committees. A companion paper examined four major theories in social science: positivism, interpretivism, critical theory and functionalism, which can encourage legalistic ethics theories or practical living bioethics, which aims for theory–practice congruence. This paper develops the legalistic or living bioethics themes by relating the four theories to clinical ethics committee members’ reported aims and practices and approaches towards efficiency, power, intimidation, justice, equality and children’s interests and rights. Different approaches to framing ethical questions are also considered. Being aware of the four theories’ influence can help when seeking to understand and possibly change clinical ethics committee routines. The paper is not a research report but is informed by a recent study in two London paediatric cardiac units. Forty-five practitioners and related experts were interviewed, including eight members of ethics committees, about the work of informing, preparing and supporting families during the extended process of consent to children’s elective heart surgery. The mosaic of multidisciplinary teamwork is reported in a series of papers about each profession, including this one on bioethics and law and clinical ethics committees’ influence on clinical practice. The qualitative social research was funded by the British Heart Foundation, in order that more may be known about the perioperative views and needs of all concerned. Questions included how disputes can be avoided, how high ethical standards and respectful cooperation between staff and families can be encouraged, and how minors’ consent or refusal may be respected, with the support of clinical ethics committees.

## Introduction

This discussion paper considers seldom recognised theories that influence the work of clinical ethics committees (CECs). The four theories or main traditions in social science are positivism, interpretivism, critical theory and functionalism. The traditions encourage partly outdated legalistic bioethics theories or, in contrast, practical living bioethics that aims for theory–practice congruence. This paper briefly summarises ideas from a companion paper^1^ in order to extend the discussion. The four theories have differing approaches to efficiency, power, intimidation, justice, equality and children's interests and rights. Different approaches to framing ethical questions are also considered.

This paper is informed by a study in two London paediatric cardiac units.^2^ Forty-five practitioners and related experts were interviewed, with their informed, written consent, about the work of informing, preparing and supporting families during the extended process of consent to children's elective heart surgery. The mosaic of multidisciplinary teamwork is reported in a series of papers about each specialty, including this one on bioethics and law. Interviewees included eight current or former members of ethics committees, and other healthcare professionals who were studying or teaching ethics. Interviewees described the influence on clinical practice of CECs, when assisting with specific ethical dilemmas, and when CEC members apply ethics in their everyday practice and influence their colleagues. One consultant, for example, worked in palliative care ‘holistic care of anyone with a life-limiting or life-threatening illness’, informed by expertise in ethics and in mediation. Palliative care involves a complex balance of support for all efforts ‘to maximise patient’s health’ while accompanying children on ‘that journey when life is limited’ (45).

The qualitative social research was funded by the British Heart Foundation, in order that more may be known about the perioperative views and needs of all concerned. Questions included how disputes can be avoided, how high ethical standards and respectful cooperation between staff and families can be encouraged, and minors’ consent or refusal be respected, with the support of CECs.

The research methods included: literature reviews and a systematic review^3^; observations in two London children's hospitals in the clinics, wards and medical meetings; audio-recorded and transcribed interviews with 45 healthcare professionals and related experts with their written informed consent, discussions with a multi-disciplinary advisory group, thematic qualitative data analysis and detailed exchanges between the co-authors. Contact with participants was by ‘phone and online only, from March 2020, and data-collecting was severely reduced by the pandemic. Interviews with children aged 6 to 15 years, and their parents, about their elective heart surgery are reported in other papers’.

Table 1 lists the interviewees quoted, their number and specialty.

**Table 1.** Quoted interviewees’ number and specialty.

Interviewee's number	Interviewees’ specialties
4	Chaplain/ethics committee member
18	Consultant anaesthetist/ethics committee member
19	Consultant surgeon/ethics committee member/member of a hospital directorate
26	Consultant surgeon/ethics committee member
30	Consultant intensivist/ethics committee member
34	Youth officer at children's heart information and support charity
35	Mediator/consultant paediatrician
43	Ethicist/ethics committee member/member of a hospital directorate
44	Lawyer/ethics committee member/member of a hospital directorate
45	Palliative care consultant/ethics committee member

Note: Some interviewees had a second or third present or previous role.

## Four traditions in social science

This section summarises a longer explanation in the companion paper,^1^ in order to provide background information for this paper. Four traditions in social science are positivism, interpretivism, critical theory^4^ and functionalism.^5^

*Positivists* work with predictable, measurable laws and systems through surveys and randomised controlled trials in the most highly funded and trusted version of social science. In ethics, deontology offers methods of analysis that echo positivism's definitive and predictable laws as principles. Utilitarianism relies on positivist evidence and methods of measuring and evaluation.

*Interpretivists* recognise great differences between the natural and social sciences. They observe and interview people, usually in small, detailed samples, about their personal views and experiences, often reported in case studies. Unlike factual positivism, interpretivism sees everything as socially constructed, contingent and often diverse. Interpretivism aligns with bioethical analysis of differing views, values and analyses, and with detailed case studies.

Both these traditions measure and describe social life, and perhaps aim to make it more efficient.

*Critical theorists* also describe and measure social life, but aim to examine and explain underlying causes. They work for a major change in how decisions, resources and power can be controlled and shared more equally.^6^ This aligns with radical ethics, feminist ethics and other ethics concerned to change the world.^7^

*Functionalists*, in contrast to critical theorists, hold conservative beliefs that society generally functions for the greater good of all, and need only become more efficient. In aiming to promote efficiency, rather than to challenge or change present systems, positivists tend to favour functionalism, which is mainly discussed in the positivist section.

The traditions involve different views about ethics. Positivists, functionalists and many interpretivists aim for value-free and emotion-free objectivity. They tend to out-source ethics into the research ethics committee review system. Critical theorists, however, contend that all social life and research are imbued with moral meaning, and social facts cannot be separated from values.^8^ They believe objectivity means being fair and open-minded, but not being value-free or neutral, which is impossible on moral questions such as about social justice. Like critical bioethicists, they recognise that we all defend certain interests and critique others, and therefore we need to be as transparent and critically reflexive about our values as possible. To functionalists, justice involves observing present laws.^9^ Critical theorists and critical bioethicists, however, seek to promote justice by challenging and changing inequalities and oppressions and promoting human rights.

The next three sections consider how CECs may be influenced by positivist and functionalist, or interpretive, or critical approaches. Interviewees did not mention the four theories. We have grouped and discussed their views in relation to the theories in order to show how it can be helpful to be aware of the theories’ influence, strengths and limitations, when seeking to understand and possibly change CEC routines.

## Positivism and functionalism in CECs

CECs review examples of patients’ care and offer advice when hospital staff report having questions or difficulties. CECs are not in every institution but are gradually spreading around Britain's hospitals. Most members are hospital staff but, like research ethics committees, CECs have a few lay members. Some have expert ethicists and legal members. Their work is described further in this section. All committees aim for responsible, expert efficiency and compassion, and they all combine positivism with interpretivism, though with different emphases.

CECs with a mainly positivist functionalist position draw on strong medico-legal bioethical traditions. One children's hospital CEC devotes an hour to each case at monthly meetings involving about 15 of the total 24 CEC members. In ∼90% of cases, parents and occasionally children attend this CEC, during the middle 20 min. Practitioners who work with the family attend for the whole meeting. There is time talking with the family before and after the meeting. The CEC also does rapid reviews, when three to six members meet the family, and it reviews cases referred by other hospitals, encouraging them to set up their own CEC. About 50 cases a year are reviewed.

The CEC chair (30), when interviewed, spoke of the challenge of aiming to be efficient and unintimidating.

We make the meeting as unintimidating as we can but, at the same time, it has to be a process where clinicians get to discuss the case as well as the family really. We have struggled with that a bit sometimes about what the right approach is … We [provide] a consensus forum … developing … something that [everyone] involved can say, ‘Yes, okay. It might not be what I thought was the best thing but I think this is an okay option’ … We think about the best interests of the child and try and get the child's 360° existence in there; the social aspects; the faith aspects; the cultural stuff but also the family dynamics … We’ve much more become a support group with complex ethical issues for children and families but also clinicians.

It is not easy for families to suddenly join a meeting of experts and briefly present their complex case, though this is possible. A chaplain and CEC member (4) said one child who wanted to attend the CEC was asked, ‘Why do you want this treatment?’ He said, ‘I want to get better. I want to go to school. I want to live my life … ’ That had such an impact on the committee and everybody else who were struggling to make that decision’*.* Working in parallel as a bioethics centre, CEC members run teaching and training in different hospitals, do primary research, write papers, run staff support against moral distress, moral disquiet and trauma, and aim to listen to everyone with a concern. They are part of the hospital wellbeing support group helping with difficult ethical or emotional cases. One of their measures of success is when families say the CEC made them realise ‘how much the institution values my child’ (30), with members aiming to make parents feel part of their meetings.

The great benefits of modern medicine raise high hopes. When these cannot be fulfilled, questions arise about whether withholding or withdrawal of treatment is in the child's best interests. In one leading children's hospital, over 3 years there were 203 such cases, and in 186 of them, the parents and doctors concerned agreed after discussions to withdraw treatment.^10^ In 17 cases there were continuing disagreements, which can prolong treatment that may cause only suffering for the child, and moral distress for the staff, while the disagreement may become intractable when parents refuse to give up hope.^11^ Parents have a greater share in discussion when doctors offer options than when they make recommendations.^12^

Difficulties with referral of cases to committees or the courts include concern that they may undermine doctor–patient relationships and autonomy.^13^ Hopes that CECs are better than law courts at resolving prolonged disagreements are not yet supported by research evidence.^14^ CECs in all-age hospitals have been criticised for their lack of paediatric expertise.^15^ There is also uncertainty about who should be CEC members, what expertise and training are needed, and how CECs should be constituted and evaluated.^14^ CECs analyse and clarify disagreements, and identify the relevant values, common ground and the child's best interests, but they can only advise, not make decisions or resolve disputes.^16^ Some lawyers consider that ‘best interests is an empty mantra’ and in intractable disputes ‘there may be no right answer’.^17^ Besides individual patients’ needs, doctors, CECs and the courts may take account of economic and effectiveness criteria. These include the public interest, just distribution of resources, and best use of scarce resources that appear not to benefit the child who is having ‘futile treatment’.^18,19^

These problems add to pressures on CECs to respect positivist and functionalist standards: informed medical debate of a standard to satisfy clinicians; efficient productive use of time and hospital resources; support for practitioners’ effective management of cases, and for the hospital's good reputation. CECs want to avoid their advice being criticised by judges,^20^ such as for not listening enough to the families, though the need for this is disputed.^21^ Some CECs involve leading lawyers (30), whereas others have lawyer members but leave legal expertise to the Trust's legal department and are guided more by ethical principles (45). There is concern that CECs may predominantly speak for professionals more than families. They could be ‘a bit more human and a bit less, “We’re a professional body’” (35).

One interviewee (44) admired the ‘intellectual firepower’ of a CEC, but another preferred ethics to be centred more in clinical team discussions, less outsourced to bureaucratic CECs, which can feel like

a hoop through which you have to jump, often a flaming hoop [when CEC members] tend to be very bright … it can be an incredibly intimidating experience for even the most senior people [and] takes an awful lot of preparation [with often inconclusive results that say,] ‘You have to make your own mind up on the basis of what we’ve discussed’, which actually doesn't take you a lot further forward but makes you feel worse (19).

This senior surgeon had experienced in another hospital that

it's increasingly clear that huge numbers of people don't feel comfortable in speaking up in when they see the most awful decision being taken. And a chairman … has to be quite robust in order to give space for people to be asked questions … not calling for knowledge, it's asking for feelings. And when you break through that barrier, people are often very wise. But … if you don't ask, you will be dominated by the people who make quick decisions [when meetings have] become much more closed to the front row … the others aren't getting a look in unless they’re very assertive (19).

An anaesthetist and CEC member agreed.

People often turn to us, wanting us to make decisions, and yet we have kind of a slightly weird position in that we’re not decision makers, we’re here to kind of … help you go through the right processes and make sure you’ve thought about the right things and we’ll offer our opinion, but it's not our decision to make [which] … is not what people are expecting of us … I think there were some [families] that found it very intimidating at first because there was just too many people involved … particularly with withdrawal of care cases, they felt like they were being bullied (18).

CECs can support doctors who have made a controversial decision, after they have all agreed it is the best possible decision, to help them to ‘stop worrying, and so to improve their concentration and general effectiveness’ (26).

Leading hospitals have two vital and partly contradictory main aims: to provide compassionate care for individual patients, and to ‘push the boundaries of what is medically possible’ through ‘pioneering research to discover and improve treatments and find cures and better treatment for life limiting and life-threatening conditions’ that might benefit many future patients.^22,23^ CECs are at the heart of resolving tensions between these aims that involve such suffering for children and parents and for practitioners.^24^

Hospital communications, public relations and fundraising departments do much good. Yet they can increase the problems in these cases. It is harder to convince parents that no more can be done if they are surrounded by displays and fundraising appeals emphasising that this is an amazing pioneering hospital, that researchers keep breaking through new frontiers, and all families feel overwhelming gratitude. CECs may be drawn into this programme of highlighting success. For example, ‘Making decisions to limit treatment or to try very experimental treatments in children can carry a great burden; the CEC helps all those involved … to know they’ve made the best decision in the best way for the child’.^25^

The CEC's innovative work in engaging children and families in discussions about the most difficult healthcare decisions supports [the hospital's] specific objective to consistently deliver an excellent experience that exceeds our patient, family and referrers’ expectations. It also meets the government's vision for shared decision making in healthcare: ‘No decision about me, without me’.^26^

In win-lose disputes, it is not possible to satisfy or exceed everyone's expectations and the exacting standard seems unhelpful. The phrase ‘no decision about me without me’ is complicated when so many children are too young, or too ill, or are deemed to be too immature to share in decision-making. Emphasising decisive action may constrain options to refrain from acting, to watch and wait, to exercise scientific caution and scepticism, and do no harm. Although CECs offer advice, not decisions, practitioners or families desperate to find a solution may perceive the advice to be a decision, an edict. If CECs are assessed on how promptly they deal with cases, it can be harder for them to hold back in non-urgent cases and leave time for parents and children to work through their moral journey. Families may be moving from doubt towards conviction and consent to treatment. Alternatively they may need to make the harder journey, from certainty that the child must be treated, through slowly growing doubt and fear of doing harm, towards faith that withholding treatment is in the child's best interests. The child's ‘best interests’ may be assumed to be best understood by expert adults who dismiss children's views if they disagree.

## Interpretivism and CECs

Children as a group are uniquely unrepresented on ethics committees, and adult members therefore have to make extra efforts to understand children's views and interests. In bioethics reports,^27,28^ occasionally written with children,^29^ there is now greater attention to informing and involving children in decision-making. During the 1980s, lay members joined medical ethics and advisory committees, and patients’ views gained new acceptance.^30^ In one example, British paediatric research ethics guidelines, written in 1980 by paediatricians, set the taking of blood samples as a ‘minimal risk’. Researchers such as epidemiologists working in schools with hundreds of children were not required to inform them or their parents or request their consent.^31^ Revised mainly by four lay members of the ethics advisory committee in 1992, the new guidelines moved from the standard of positivist generalisation to one of interpretive awareness. ‘A procedure which does not bother one child arouses severe distress in another … Many children fear needles and for them low rather than minimal risks are often incurred by injections and venepuncture’.^32^ The revised ‘low risk’ assessment required researchers to request informed consent and respect refusal. The report caused long debates and was not published in a journal until 2000.^33^ CECs benefit from independent lay members who are real patient representatives, who have experienced difficulties and disadvantages, and speak for ‘ordinary patients’. They are preferably recruited through local communities not through hospital networks of retired and non-medical professionals (43).

In the interpretive approach, to promote respect and equality CECs are less formal and bureaucratic. Relations between different professions and grades of staff are now generally far more equal than in the past with much less of the ‘old school consultant’ (45) though there are still hierarchies. Interpretive CECs take extra time to explore members’ perceptions, and to question, for example socially constructed power relations that positivists may take for granted. It can be easier for CEC members to agree with the clinicians, who are colleagues they may have to work with again in the future, than to agree with the mainly transient ‘lay’ patients and their versions of knowledge. CECs need to be distinct from clinical governance, managerial surveillance, policy and politicking (43). Yet to challenge the hospital systems that exist can take much reflection and effort. Managing disagreement is a key task for CECs and much depends on the chair's skill in sensitively managing negotiations and relationships, which can become tense and emotional (43). If discussions are to be fair, clearly reasoned, non-judgemental, exploratory and open, in an advisory spirit, members have to be conscious of how hierarchies and possible conflicts of interests between the professional groups and the patients influence their discussions.

Clinical ethics is emotional, concerning cultures, values and norms that staff and families identify with and care about passionately. Young children's ‘discernable wishes and feelings’^34^ may involve their deep ‘carings’.^35^ To support the patient's wishes can involve all CEC members reflecting on the interpretations and values of all the individuals and groups in a particular case. They need to think about disadvantage and vulnerability in all their forms, rather than utilitarian calculation of the greater good for the greatest number, or the application of abstract principles. There is a ‘walking alongside people’ in their infinite variety, and seeing how formal policy, guidelines and frameworks stand or fall by their related human interaction. CECs may be encouraged to put the patient or family at the centre and move away from their own moral assumptions and preferences in a deliberate, structured way (43).

## Critical theory and CECs

An example from sociology of critical theory that is relevant to consent and to discussions in CEC is Jürgen Habermas's ‘ideal speech situation’. This direct communication, that reaches shared understanding through truth, trust and shared goals,^36^ is one way of describing consent. Habermas's critical theory divided society into two influential arenas: system and lifeworld. In the public system of states, markets and other formal organisations, direct communication is distorted and driven by political and economic interests. In contrast, the lifeworld of informal family, community and voluntary association encourages ideal speech. Habermas believed the system constantly disables and colonises the lifeworld: what was once personal becomes publicly organised. For example, large paediatric cardiac teams and CECs partly replace the more individual doctor–patient relations of a few decades ago. The large teams demonstrate decades of successfully developing cardiac subspecialties with their rapidly expanding knowledge and skills, and reflect the need for decision making by consensus, though they can complicate consistent personal communication.

Habermas considered that citizens sense when institutions are just and benevolent, work in citizens’ best interests, and deserve their support and loyalty. The NHS is one example, and CECs share its great strengths but also the challenges of large systems that serve many diverse individuals. CECs safeguard the good management, reputation and funding of their hospital system and the need to prevent costly litigation. Some CECs do not meet patients, hoping to be objectively detached, whereas others interview patients or their representatives. Yet CECs’ efforts to hear children's and parents’ views are hampered in several ways. Complicated personal lifeworld experiences of illness and treatment lose meaning and value if they are over-reduced into the system's clinical terms and reports.

In bioethics, the professional or expert voice is generally privileged at the expense of meaningful personal exchange and dialogue,^37^ and of careful listening to families’ accounts. In large meetings, patients feel less able to express their complicated views fully. Families and CEC lay members may feel intimidated and silenced, or only able to say what they believe is acceptable to the CEC and not their real insights, as research about lay members of other public bodies has found.^38,39^

Critical theory examines inequalities of power, authority and control over events and resources, and advocates greater equality. It was particularly discussed by interviewee 43 who cited the critical philosopher Miranda Fricker. Fricker^40^ proposed that epistemic injustice involves two kinds of unfairness related to knowledge. First, in testimonial injustice, genuinely lay people (not the quite high-status people often appointed to CECs) are ignored or disbelieved because of their lay status, and their identity if they belong to a disadvantaged or minority group.^41,42^ These ‘asymmetries, dependencies, and power relations can increase the vulnerability of patients to epistemic injustice’.^43^ Second, in hermeneutic or interpretive injustice, CECs can be locked into cycles of misunderstanding, mistakes, impatience or embarrassment.^44^ Lay members may routinely be interrupted, talked over, dismissed or challenged by higher status members. Leading members tend to insist on ‘high’, meaning academic-style, standards of debate. They may subconsciously expect the lay person to fail, and instinctively behave in ways that initiate and reinforce that failure. The more powerful members are not intentionally hostile, but to lay and other lower status members they can seem hostile if they exert their senior status, often subconsciously, such as by glancing at a mobile phone during discussions. Uniforms can stress CEC members’ professional status and weaken an ethos of moral equality among all CEC members. Subconscious behaviours are also influenced by medical and legal authority, gendered and racialised relations, and the format of meetings, seating arrangements, the needs of the hospital and the NHS, and many other system structures.^45^ The CEC chair has to be extremely skilful, alert to absences, gaps and inequalities, and to preventing or unlocking negative interactions while maintaining everyone's trust and goodwill (43).^46^

Power imbalances can partly be redressed by limiting membership to around 12 members. One member or a small subgroup may work closely with the patient and family. Although social workers and play specialists tend to gain families’ confidence, they may not be able to inform and influence the CEC's views as effectively as more authoritative members can. Some CECs invite patients to send a written statement, though this can exclude many who are unable or unwilling to write about their views. After collecting patients’ views, CECs usually later discuss them in the person's absence. The views might then be misinterpreted, so that ‘listening to patients’ risks becoming a rather empty ritual, which may be used by CECs to claim undue authority for their eventual conclusion. With children's cases, CECs need child advocate members, experienced in listening to younger and older children, and expert in up-to-date research about children's rights and competence.

CECs can become part of ongoing disagreements between unequal groups.^47^ They cannot override the clinician's ultimate responsibility, and even the law courts can only authorise clinical action, not enforce it. CECs can genuinely engage with clinicians and respect their preferences, but CECs may then explain why they are not persuaded by them (43). They may raise questions that help clinicians to think in a slightly different way, suggest a new option or priority, return to a value that was overlooked earlier, or suggest delaying a non-urgent decision. They can involve new people, or conversations, new literature or other resources. Using interpretive and critical insights, CEC members may work to unlock a block or shift a blind spot in communication or relationships so that clinicians may then feel ethically more comfortable in coming to a decision they initially rejected. These approaches to ethics can filter into clinical practice. After one CEC meeting of intensive discussion, a consultant member said, ‘As always, I’m changed and I’m going to take what I’ve heard today out into my clinical work’ (43). An example follows of these approaches when interpretive and critical bioethics serves rather than leads.

## Framing an ethical question

Living bioethics seeks theory–practice congruence or consistency. The following example combines thinking about ethics in interpretive and critical ways with practical ethics in everyday life. ‘Jasmine’ aged 14 years wants to refuse high-risk heart surgery that she believes will do little to relieve her severe health problems. Her parents insist she should have surgery. In such a highly sensitive case, how can CECs contribute? One interviewee (43) described an interpretive critical approach.

The CEC could ask what those concerned perceive the ethical question to be.^47^ Questions can be framed in several ways, such as a refusal of treatment being a conventional clash between a young person and the caring adults, or an effect of temporary depression (34). Or the CEC might ask, ‘What does good care look like for Jasmine?’ or ‘What is perceived to be at stake, and by whom? What is the ethical question? Is it A or B, A and B, or A, B and C?’ The question might be, ‘What would the virtuous paediatric team do for Jasmine?’ Or ‘What types of knowledge are involved?’ Besides medical knowledge, there are questions about Jasmine's lived experience, values and identity, her dignity and quality as well as quantity of life. The CEC could map all those questions and attend as much as needed to each framing question. Members would listen to Jasmine and give equal and maybe more weight to her view than to the clinical team's views. Some nurses and junior doctors who spend more time with Jasmine might have different views from more senior practitioners.

The CEC would question the timing, what choices have to be made, when and where. Are they urgent? Is potential transfer to palliative care rejected by some practitioners as ‘giving up’ rather than as ‘good care’? The analysis might take a whole meeting or two or three meetings with discussions with the clinical team. The CEC would offer continuing support, as a mediator between the team and the family if needed, though always with everybody thinking about the focus and purpose of each conversation. The questioning can put a mirror up to assumptions that are often embedded in the system and try to make space for imagining Jasmine's viewpoint. Imagination and empathy are as useful in ethics as analysis, to open different views. More than being guided by the child's age, adults need to attend to all the ways of knowing, thinking, feeling and being in the world, Jasmine's values, identity and connections. Responses to these depend not on being detached, expert and powerful, as health professionals are trained to be, but on wisdom, discernment and basic ways of being that can manage uncertainty, emotion, complexity and pressure.^47^

Ethics degree courses emphasise knowledge, which brings power and authority, as for example in the four principles model.^48^ Yet while medical knowledge involves mainly predictable, calculated cause-effect processes, ethics involves many known and unknown causal influences, subjective unpredictable human agents, complex relationships, contexts and histories, and dilemmas without clear resolution. Ethics therefore tends to involve greater uncertainty and broader open inquiry than medicine. Degree courses could attend more to tentative interpretive ethics, the feminist ethics of care, virtue ethics, and the actual practices and relationships involved (43).

Some CECs have no members with obvious ethics expertise, although that expertise is a complicated concept if it seems to claim superior moral knowledge and skills. Clinical ethics gains from people with different approaches, training and experience all working together. A wise approach in bioethics can be the philosopher paediatrician John Locke's humble aim to be the under-labourer, ‘clearing the ground a little, and removing some of the rubbish that lies in the way to knowledge’,^49^ as this paper attempts to do.

Interpretive chairing of a CEC can be difficult emotionally and practically, in terms of being inclusive, balancing people's emotions in the room, and holding and containing often passionate disagreements.^50^ The chair has to provide a structure that is safe, so that members need not be defensive. This can take a great deal of concentrated energy and quiet confidence (43). Relational ethics is helpful when it works through inclusive methods and is affective as well as effective.

Practical ethics involves virtuous habits and is always work in progress. When challenged and threatened by someone you find difficult, to act well can be the reality that makes or breaks clinical ethics (43). This invokes Bion's psychoanalytic theory of therapeutic containing, when the mother allows her baby to express overwhelming emotions of distress, fear or anger.^51^ She receives these unwanted projections from the baby and contains and processes them, then returns the experience to the baby in a modified manageable form. Winnicot^52^ analysed how mothers teach their children gradually to hold and cope with powerful emotions and needs. Like therapists, virtue ethicists listen to outbursts of anger or distress, then work to contain and interpret them into forms that CEC members, and the patients and practitioners they serve, can accept and develop. Instead of power and authority, the aim of humility and courage is to be open to not-knowing, or to be aware when mistaken. This can feel threatening to members. They need a containing environment if humility and uncertainty can flourish, and even be celebrated as virtues in open discussions (43). Medical ethics can be

a way of working that fosters opposition when healthcare is, in general, an uncertain and collaborative endeavour. Adopting and arguing for a position is considered integral, even essential, to good medical ethics. The alternative is often characterised as confused or weak. Yet, not taking a ‘position’ recognises false divides and allows for listening, dialogue and learning.^50^

One interviewee (43) discussed how the framing of questions, considered earlier, helps to deepen CEC discussions, as can a ‘shadow’ meeting to discuss a hypothetical case. Members then analyse their discussions and review: the hierarchies and power structures; how members respected certain individuals and ideas and dismissed others; how their thinking, assumptions, working methods and discussions might become more open and equal; how the meeting room might be more welcoming; who refers cases and how these are framed; how success is defined and measured. Visitors might be invited to comment as critical friends, and members might observe other CEC meetings. There also could be ‘rehearsals’ about how CECs manage difficult choices and conversations, or communicate unwelcome news, how they cope with angry hostile discussion, or tolerate emotional discomfort. Members may be more worried, not about making a choice, but about enacting the choice, and rehearsals can increase their confidence. Rather than the positivism that wholly supports evidence-based medical decisions, critical interpretivism gives ‘due weight’^53^ to the views of the child and takes account of her ‘wishes and feelings’.^54^

## Conclusion: Ways forward in living bioethics concerned with children

The living ethics of justice, care and respect for children and their consent depends on both theory and practice, contexts and relationships. CEC members’ polite relationships with families, and sometimes with their lay and lower status members and, indeed, doctor–patient relations generally, can be undermined by unseen influences. These range from the history of ethics with its emphasis on an imagined emotion-free adult-centric rationality (discussed in the companion paper^1^); misleading developmental psychology that underestimates children; legal and financial pressures on the need for consent to be an adult contract; management systems and daily routines in healthcare that can intimidate and cause stress and anxiety for families and staff; social inequalities of class, race, gender and age. Unless these are actively attended to, their subconscious and practical effects can be still more powerful.

Each approach to ethics has strengths and weaknesses. Positivist concern with evidence and reason is vital for informed consent. Interpretive approaches are needed to help everyone to understand the relevant knowledge and the range of views concerned more widely and deeply, and to respect the moral emotions of trust and voluntariness. Yet positivist approaches can be narrowly exclusive. Interpretivist approaches risk seeming vague and slow when families and staff urgently need help, and unpaid CEC members have much else to do. Virtue ethics offers practical ways to work with these challenges. Yet it makes great demands on practitioners, who already have to satisfy daunting professional standards often in under-resourced and not wholly supportive contexts. This returns to the need to work on raising standards in contexts as well as with individuals, in awareness of underlying theories and assumptions, so that positivist, interpretive and critical approaches can be combined in the most effective ways.

One CEC chair (43) saw a criterion for success in meetings and encounters as resonance. This brings change, new insights, questions and value to clinical care, though it is perceived differently by different people. When pragmatic practitioners pressure CECs to produce prompt summaries, decisions and action, the questions can start from their viewpoint and constraints, but ask, ‘Why and what might the implications of that be?’ Then, ‘What would optimum care look like, when a decision cannot be comfortable or ideal? How best could it be explained to somebody who does not understand the choice that has been made? How might it be justified to someone who disagrees with it?’ CECs benefit when all members feel able to ask challenging questions and do not leave this to a few confident members. The method is not to assume the solution, but to be open to other perspectives that are approached in many different ways, as this paper has aimed to show. Living ethics involves being and doing ethics as well as knowing and talking about it.
